# Echoes from Sensory Entrainment in Auditory Working Memory for Pitch

**DOI:** 10.3390/brainsci14080792

**Published:** 2024-08-07

**Authors:** Matthew G. Wisniewski

**Affiliations:** Department of Psychological Sciences, Kansas State University, Manhattan, KS 66506, USA; mgwisniewski@ksu.edu

**Keywords:** auditory memory, inhibition timing, neural entrainment, oscillation, auditory rhythms

## Abstract

Ongoing neural oscillations reflect cycles of excitation and inhibition in local neural populations, with individual neurons being more or less likely to fire depending upon the oscillatory phase. As a result, the oscillations could determine whether or not a sound is perceived and/or whether its neural representation enters into later processing stages. While empirical support for this idea has come from sound detection studies, large gaps in knowledge still exist regarding memory for sound events. In the current study, it was investigated how sensory entrainment impacts the fidelity of working memory representations for pitch. In two separate experiments, an 8 Hz amplitude modulated (AM) entraining stimulus was presented prior to a multitone complex having an *f*_0_ between 270 and 715 Hz. This “target” sound could be presented at phases from 0 to 2π radians in relation to the previous AM. After a retention interval of 4 s (Experiment 1; *n* = 26) or 2 s (Experiment 2; *n* = 28), listeners were tasked to reproduce the target sound’s pitch by moving their finger along the horizontal axis of a response pad. It was hypothesized that if entrainment modulates auditory working memory fidelity, reproductions of a target’s pitch would be more accurate and precise when targets were presented in phase with the entrainment. Cosine fits of the average data for both experiments showed a significant entrainment “echo” in the accuracy of pitch matches. There was no apparent echo in the matching precision. Fitting of the individual data accuracy showed that the optimal phase was consistent across individuals, aligning near the next AM peak had the AM continued. The results show that sensory entrainment modulates auditory working memory in addition to stimulus detection, consistent with the proposal that ongoing neural oscillatory activity modulates higher-order auditory processes.

## 1. Introduction

A growing number of theoretical works are proposing that ongoing oscillatory brain states drive listening successes (e.g., segregation of speech into meaningful units [1]) and failures (e.g., distorted intensity perception in schizophrenia [2]). It is generally posited that these oscillations reflect cycles of excitation and inhibition in local neural populations, with individual neurons being more or less likely to fire depending upon the ongoing phase of oscillatory cycles [2,3,4,5,6]. As a result, ongoing oscillations determine whether or not a near threshold sound is perceived and/or whether a sound’s neural representation enters into later processing stages [3,5,7,8]. While empirical support for these ideas is also growing, large gaps in knowledge still exist. In particular, little is known about how ongoing oscillations impact the quality of later memory for sound events.

In large part, research in auditory science has focused on how oscillations impact sound detection. When oscillations are entrained at a particular rate through repetitive stimulation, this entrainment modulates both neural oscillatory phase and the detectability of a presented sound. For instance, one recent study found that entrainment “echoes” could be seen as cyclic fluctuations in the detection of pure tone signals after the presentation of an amplitude modulated (AM) entraining sound [9]. These echoes showed up most prominently in the 8 Hz range with the best detection aligning to the peak of where the entraining stimulus would have been if it had continued (see Experiment 4; [9]). Henry and Obleser [10] presented listeners with a 3 Hz frequency modulated entraining stimulus with randomly occurring gaps in the sound near listeners’ individualized detection thresholds for gap duration. Gap detection performance was cyclically modulated and also more in line with neural 3 Hz EEG phase than stimulus 3 Hz phase across subjects (also, see [11]). This suggests more neural oscillation dependence than stimulus oscillation dependence for detection, an important finding, since entrainment was continuous in that study (i.e., cyclic performance could potentially be explained by cyclic acoustics). Several other studies have revealed similar findings (for review, see [12,13]).

Investigations involving auditory tasks more complex than detection are needed to evaluate current ideas about the role of oscillations in audition. For instance, in proposing a “unifying account” of neural entrainment, Lakatos et al. [2] describe entrainment as supramodal to include not only sensory areas but also regions involved in memory. In their account, oscillations in sensory areas can entrain oscillations in higher order cortical structures important for memory operations (also, see [14]). This, in turn, could establish functional connectivity between sensory representations and later processing stages. In a similar vein, a recent attempt at reconciling the popular embedded processes model of attention and memory [15] with theory on the functional roles for neural oscillations proposes that oscillations control the attentional selection of items that are stored in memory [8]. According to those authors, oscillations could determine what is or is not activated or updated in memory. On the empirical end, Hansen et al. [16] examined the relationship between the spontaneous prestimulus EEG phase and performance in a pitch sequence identification task requiring the memory comparison of successive tones of varying pitch. They found that correct and incorrect trials had opposing phases within the alpha range (strongest at around 8 Hz) just prior to sound onset (also, see [17,18,19]). Those authors proposed that the previous null effects found for spontaneous oscillations in auditory studies (e.g., [20]) could be because oscillations more strongly impact higher-level cortically dependent auditory processes than those necessary for simple detection tasks; the computations for the latter could be conducted largely at a subcortical level. Research is needed for tasks employing higher working memory demands to adequately assess these ideas.

Here, it was investigated how entrainment impacts the fidelity of working memory for pitch. In two separate experiments, an 8 Hz amplitude modulated (AM) entraining stimulus was presented prior to the presentation of a multitone complex having an *f_0_* between 270 and 711 Hz. This “target” sound could be presented at phases from 0 to 2π radians in relation to the previous AM. After a retention interval of 4 s (Experiment 1) or 2 s (Experiment 2), listeners were tasked to reproduce the target sound’s pitch by moving their finger along the horizontal axis of a response pad. Thus, the task requires both the retention and manipulation aspects of auditory working memory. Based on the theoretical and empirical work discussed above, it was expected that reproductions of a target’s pitch would be better when targets were presented in phase with the entrainment (cf. [9]). The results are discussed in regard to their theoretical relevance and the employment of similar tasks to advance knowledge in this area.

## 2. Experiment 1

### 2.1. Materials and Methods

#### 2.1.1. Listeners 

The listeners were 26 individuals (19 female) participating in exchange for credit in psychology courses at Kansas State University. The average age of the listeners was 19.3 years (*SD* = 1.6). All reported normal hearing and signed an informed consent form. The procedures were approved by Kansas State University’s institutional review board.

#### 2.1.2. Equipment

**Figure 1:**
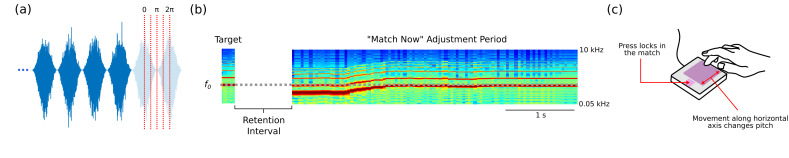
Task details for Experiment 1. (**a**) Waveform for the end of an entraining AM stimulus (dark blue) along with outlines for what the AM would have looked like if it had continued. The red lines show where the target onsets were for each phase condition. (**b**) A spectrogram example of what the matching part of the task could look like. A target sound is presented; then, there is a retention interval and finally an adjustment period in which a listener produces a sound that best matches their memory of the target. Here, the listener starts at a pitch lower than the target before adjusting to a higher pitch that is a closer match. (**c**) Depiction of the response pad on which listeners produced matches of the target.

The listeners sat in a sound-attenuating booth throughout the study (WhisperRoom, Knoxville, TN, USA). They heard sounds presented over Sennheiser HD-280 closed-back headphones (Sennheiser, Wedemark, Germany) connected to an RME UC audio interface (RME Audio, Haimhausen, Germany). They moved their finger along a pressure-sensitive Roli Lightpad Block MIDI controller (Luminary, London, UK) to match the pitch of a sound from memory (see Figure 1c). All experimental procedures were executed in MATLAB 2019a (Mathworks, Natick, MA, USA).

#### 2.1.3. Stimuli

Multitone complexes summing pure tone signals including *f*_0_, *f*_2_, *f*_4_, *f*_6_, *f*_8_, and *f*_10_ were used as stimuli. The *f*_0_ for the target sound in a trial varied randomly among 211 possibilities log-spaced between 270 Hz and 715 Hz. The level of each successive harmonic was decreased 3 dB from the previous, with overall sound level calibrated to be presented at ~70 dB HL. The target sounds were 50 ms in duration with 5 ms cosine shaped on- and offsets. White noise (2 s) was amplitude-modulated at 100% depth with a cosine wave function at 8 Hz. This AM noise stimulus was used for entrainment and presented at −8 dB in relation to the target multitone complex (relative to the max intensity).

#### 2.1.4. Task and Procedures

Figure 1 depicts the task. Each trial contained the 2 s AM noise presented just prior to a target sound, followed by a 4 s retention interval and the matching period. Listeners were instructed that the AM noise was meant to warn them that a target sound was upcoming and that their job would be to hold that target in memory for later matching. Listeners were given no instructions related to the timing of the target presentation in relation to the AM. A fixation cross was presented at the center of the computer screen for the entrainment and target presentation periods. A “match now” text prompt was presented during the matching period. In this matching period, the listener initiated a sound with an *f*_0_ from 220 Hz to 880 Hz by pressing their finger on the MIDI response pad. The *f_0_* of that initial sound was dependent upon the placement of the finger. The movement of their finger along the horizontal axis of a lit area of the response pad (purple box in Figure 1) changed the pitch of that sound. The pitch was mapped onto the horizontal axis such that a low *f*_0_ corresponded to the left portion. The *f*_0_ increased in logarithmic increments with each incremental movement to the right. Once convinced of a match, listeners could lock their selection in by pressing a separate portion of the response pad (pink square in Figure 1). Listeners had 10 s to complete this. After locking in a response, the next trial was initiated after a variable inter-trial interval (0.5–1.5 s; uniform distribution).

The critical aspect of the design was that the target could occur at phases of 0 (in phase), 0.5π, π (opposing phase), 1.5 π, and 2π (in phase) radians in relation to the next peak of the entraining AM noise (see possible target onsets in Figure 1a). The phase conditions were randomly ordered within a block of 15 trials, in which 3 trials of each phase condition were presented. There were a total of 10 blocks in the experiment. After each block, a listener was shown a scatterplot of their data (phase conditions unidentified) with a unity and a regression line so that they could continually assess their performance.

In order for listeners to become accustomed to this unique task, the experimenter initially gave a demonstration of 3–5 trials using the response pad themselves with sounds presented over speakers outside of the booth with a separate computer setup. In the demonstration, the experimenter demonstrated the range of pitch adjustments that could be made and noted the difference in the AM and the target so that the subject was sure of which stimulus to match. After this, the subject was placed in the sound booth and started the experiment. The experimenter went into the booth after the first block of trials to explain the scatterplot of the listener’s data and to query as to whether they had any misunderstandings about the task. This first block was considered a practice block and was not included in later data analyses. The whole experimental session took ~1 h.

#### 2.1.5. Measures and Statistics

Linear regression models were fit to individual listener matching data predicting the matched *f*_0_ from the target *f*_0_ for sounds occurring at each phase condition. These regression models were used to extract the accuracy and precision measures (cf. [21,22]). At the extreme ends of the adjustable *f*_0_ range (220 and 880 Hz), the absolute value of the difference in semitones between the predicted matched *f_0_* and the actual target *f*_0_ was taken. The average of these absolute errors was taken as an accuracy measure. For a measure of precision, the standard deviation of residuals around the regression line was used. Note that this is the inverse of precision; however, the term precision is used to describe this data to maintain consistency with the existing literature. 

A non-parametric permutation-based approach was taken to assess the hypothesis that auditory working memory for pitch would be modulated in phase with the entraining AM. First, a cosine function was fit to the average accuracy and precision data with maximum likelihood estimation (cf. [10]). In the equation, *y* is the dependent measure of interest, *a* and *b* are free parameters associated with cosine amplitude and bias (i.e., >0 error or SD), respectively, *c* is the phase offset, and *x* is the phase condition (in radians) in relation to the AM entraining sound. The *a* parameter was restricted to values between −∞ and 0, and *c* was fixed at 0. This was to account for the prediction that in-phase target onsets would show the lowest error (or smallest variability). The *R*^2^ of this fit was recorded for the actual data. Next, the phase condition labels were shuffled for each trial within an individual, and the fit was recomputed. This was performed for 500 iterations. The proportion of the resulting *R*^2^ values in the permutation distribution exceeding that of the actual data was considered the *p* value and assessed for significance at an alpha level of 0.05.

(1) $$y=a\times cos\left(x+c\right)+b$$

While the prediction was made that performance should be best at phase conditions aligned with the peak amplitude of the entraining stimulus, it is possible that cyclical performance could show up consistently across individuals at a different “optimal” phase and/or at different optimal phases depending upon the individual (e.g., see [10]). To evaluate this possibility, an individual’s data were fit with the same function, allowing *c* to vary as a free parameter. It was predicted that the individual *c* values should fall near 0. The non-uniformity of the resulting phase offsets was tested with a Rayleigh test (alpha = 0.05). A 95% confidence interval for the mean phase offset was generated and used to determine whether the phase was aligned to 0 as predicted. These circular statistics were conducted in the CircStat toolbox for Matlab [23]. It is also important to make clear here that although the phase condition is correlated with duration from the offset of AM, the hypothesis of a cyclic echo in memory performance means that a significant effect would not be related to this design aspect. That is, a significant cosine fit of the data cannot be accounted for simply by the linear passage of time. 

### 2.2. Results

**Figure 2:**
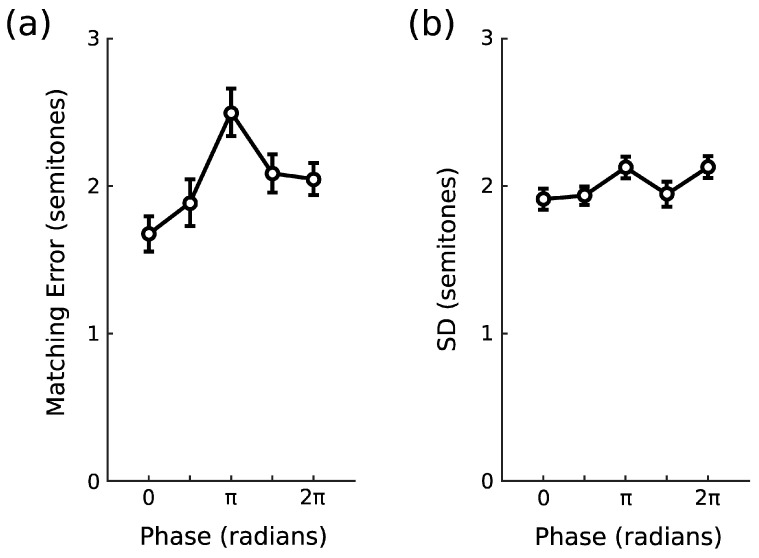
Experiment 1. (**a**) Average matching error and (**b**) standard deviation of listeners’ reproductions of the target sound at each relative phase offset from the entraining AM stimulus. The error bars show within-subject standard errors of the mean [24].

Figure 2 shows the accuracy (a) and precision (b) data. As hypothesized, there was an apparent cyclical modulation of accuracy where performance was better at phases in line with the entraining AM (0 & 2π) but worse at the opposing phase (π). This is similar to that observed by L’Hermite and Zoefel [9] in auditory detection at the same rate of AM. Statistical testing revealed the data to be better fit to the hypothesized cosine function than expected by chance, *p* = 0.048. However, there was no significance found for the same test performed on the precision data (Figure 2b), *p* > 0.20.

**Figure 3:**
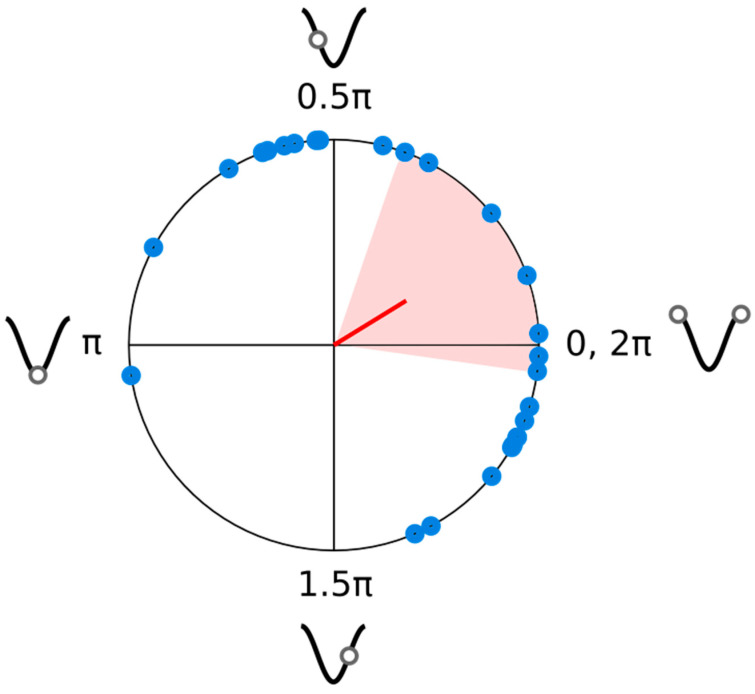
Polar plot of the individual phase offsets from the fitted cosine of the accuracy data (blue circles). The average phase vector (red line) and a 95% confidence interval for the average phase angle (shaded region) are also shown. The images beside each quadrant intersection show the points where a target would have fallen in AM, had the entraining stimulus continued.

Figure 3 shows the individual phase offsets determined from fitted accuracy data (blue circles). A Rayleigh test confirmed that the distribution of phase offsets was not uniform, Rayleigh’s *Z* = 4.46, *p* = 0.01. The mean phase offset was 0.55 radians, with a confidence interval that overlapped 0, [−0.14 1.24]. This average phase offset is consistent with the original prediction of the best performance being in line with the peak of AM had it continued for the entraining stimulus.

### 2.3. Discussion

The accuracy data from Experiment 1 suggest that entrainment cyclically modulates auditory working memory accuracy for pitch. When a to-be-encoded sound was presented in phase with the entraining AM, the matching accuracy was better than when a sound was presented out of phase. However, this was not reflected in the precision of matches as expected. In addition to a true negative result, this could potentially be due to a number of other factors. Too few trials (27 per phase condition, per subject) could have made it difficult to attain stable measures of precision. The use of a cosine-shaped AM may have produced strong individual differences in the phase locking to the sound with some locking to low-amplitude and others locking to high-amplitude portions (for a related discussion, see [10]). The lack of a precision effect could also be because the to-be-matched sound was too long in duration to show specificity to a particular phase condition. That is, at a duration of 50 ms, the target presentation extends across a swath of phases. Encompassing in-phase time points for an out-of-phase condition may have muddied any precision effects. There could have also been effects related to the entraining stimulus offset (e.g., forward masking; see [9] for discussion). Experiment 2 was conducted to address these possibilities and to test the replicability of the observed accuracy effect.

## 3. Experiment 2

Experiment 2 incorporated several changes from Experiment 1. First, the retention interval was shortened to 2 s in order to increase the number of trials that could be collected within an hour session. Second, the duration of the target was halved to limit bleeding the presentation of the target into adjacent phase bins within the 8 Hz cycle of entrainment. Lastly, the AM for the entraining stimulus employed an inverse sawtooth waveform so as to limit the potential forward masking for the early phase bins and to limit the potential variability in how individual listeners may entrain to the AM (e.g., according to the quietest or loudest portions of AM). With a sawtooth, the quietest and loudest points occur at approximately the same point in time. 

### 3.1. Materials and Methods

#### 3.1.1. Listeners

The listeners were 28 individuals (18 female) who participated in exchange for credit in psychology courses at Kansas State University. The average age of the listeners was 18.9 years (*SD* = 0.6). All reported normal hearing and signed an informed consent document. The procedures were approved by Kansas State University’s institutional review board.

#### 3.1.2. Equipment

All equipment was the same as Experiment 1.

#### 3.1.3. Stimuli

The stimulus parameters were mostly the same as Experiment 1, with the following exceptions. The target sounds were reduced in duration to 25 ms, with 5 ms on- and offsets (cosine shaped). The AM 2 s noise was inverse-sawtooth modulated (decreasing in amplitude over a cycle).

#### 3.1.4. Task and Procedures

The task and procedures were the same as Experiment 1 with the following exceptions. The retention interval duration was shortened to 2 s. The number of blocks was increased to 14 to increase the number of trials per phase condition; there were 39 per phase condition after eliminating the practice block from analysis.

#### 3.1.5. Measures and Statistics

The measures and statistics were the same as Experiment 1.

### 3.2. Results

**Figure 4:**
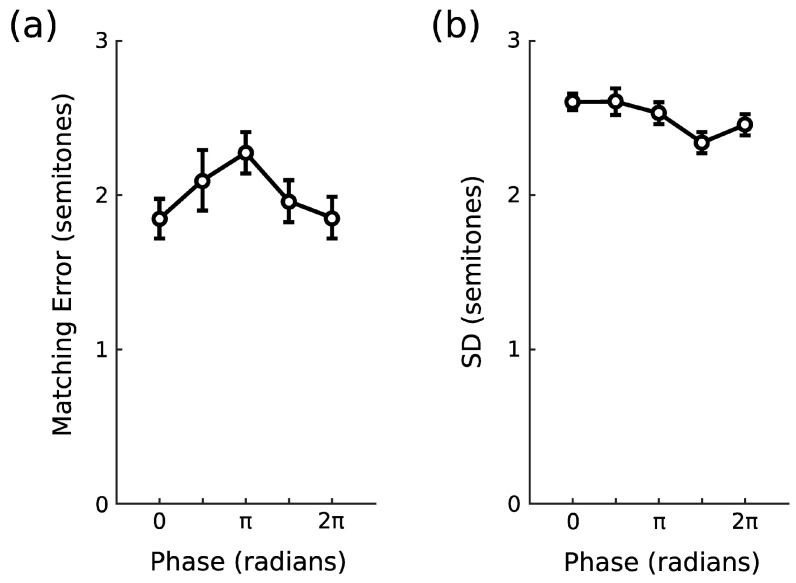
Experiment 2. (**a**) Average matching error and (**b**) standard deviation of listeners’ reproductions of the target sound at each relative phase offset from the entraining AM stimulus. The error bars show within-subject standard errors of the mean [24].

Figure 4 shows the accuracy (a) and precision (b) data from Experiment 2. The data mimic that observed in Experiment 1, with a cyclic modulation of memory accuracy after completion of the entraining AM stimulus, *p* < 0.001. The matching accuracy was best at the times aligned with the peak of the AM had it continued. The performance was worst at the trough of the entraining AM. There was not a significant effect for precision, *p* > 0.20.

**Figure 5:**
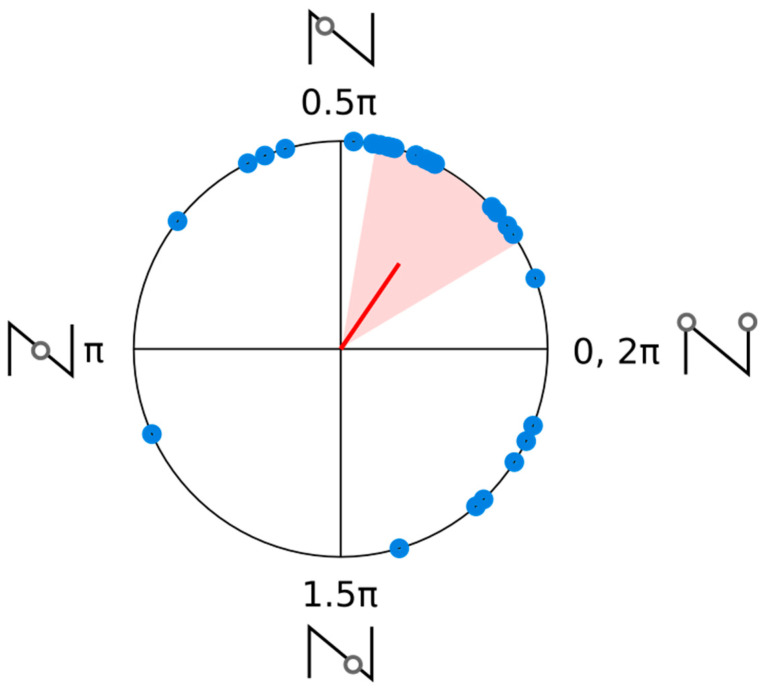
Polar plot of the individual phase offsets from the fitted cosine of the accuracy data (blue circles). The average phase vector (red line) and a 95% confidence interval for the average phase angle (shaded region) are also shown. The images beside each quadrant intersection show the points, where a target would have fallen in AM, had the entraining stimulus continued.

Figure 5 shows the individual phase offsets determined from the individual fitted data (blue circles). A Rayleigh test confirmed that the distribution of phase offsets was not uniform, Rayleigh’s *Z* = 9.39, *p* < 0.001. The mean phase offset was 0.97 radians, with a confidence interval that in this case did not overlap 0, [0.53 1.40]. However, it should be noted that this did not overlap any of the tested phases, and that the mean vector was still closer to a phase of 0 than the opposite phase (π).

### 3.3. Discussion

Experiment 2 replicated Experiment 1’s finding of the cyclical modulation of auditory working memory accuracy for pitch by an entraining AM sound. However, even after making adjustments to the paradigm, there were no apparent modulations in the pitch precision.

## 4. General Discussion

The current study evaluated the hypothesis that sensory entrainment can produce cyclic echoes in auditory memory performance. Though this hypothesis has been made by several, memory studies have taken a back seat to studies focused on detection in regard to entrainment effects. Here, individuals entrained with an AM noise stimulus at 8 Hz showed corresponding modulations in the accuracy of their pitch matching for a sound falling at different phases of the modulating oscillation. This was in an “echo”, in that it was observable after the modulating stimulus was stopped. Two experiments showed that this effect holds for changes in the target duration and AM waveform shape. There was no statistical indication of an echo in the precision to which individuals matched pitch. Nevertheless, this work is consistent with the prediction that ongoing brain oscillations modulate working memory for sounds.

Inhibition-timing views on the functional role for neural oscillations suppose that ongoing oscillatory brain states reflect pulses of inhibitory activity in the cortex such that the inputs presented in one phase will yield firing, whereas the inputs in an opposing phase will not [2,3,4,5,6]. That the current experiments revealed the modulation of auditory memory performance by 8 Hz modulation is wholly consistent with this view and can be taken to suggest that oscillations play a similar role in audition as they do in other modalities, where this hypothesis has been investigated more thoroughly. From the view of inhibition timing, a cyclic modulation was observed because entrained oscillations (presumably in the auditory cortex) modulated the firing of neurons coding for pitch information. Indeed, there has been some work in nonhumans showing that oscillations in the primary auditory cortex impact the sharpness of frequency tuning [25], which in turn could impact the accuracy and precision of sound representations in memory [8,26]. Another possibility, however, is that separate brain rhythms become entrained to the AM and, by consequence, establish a form of functional connectivity among different regions important for auditory memory processes [3,4,7,8,27]. From the perspective of these “communication through coherence” views, action potentials occurring at the optimal phase of an oscillation in an auditory area arrive at another brain area at a similar phase and are thus more likely to trigger action potentials in that area as well [4]. This could occur, for instance, in the hierarchical progression of signals from the primary auditory cortex up to the auditory responsive regions of the prefrontal cortex known to be involved in working memory for sounds [28]. 

Though the current data cannot distinguish between these different possibilities, future research that combines the current behavioral method with the recording of actual neural oscillations could potentially accomplish this. For instance, a study looking at how responses to target stimuli at different phases vary among different sources of EEG responses to the target might reveal modulations of typical auditory responses (e.g., N1 evoked potentials), later non-auditory responses (e.g., P3), or both. Further, source analyses of oscillatory activity with procedures like independent component analysis [29] could provide a window into how independent brain rhythms are impacted by sensory entrainment. Based on previous empirical work [10,11,30,31] and the notion that sensory entrainment produces supramodal effects [2], it can be expected that auditory and non-auditory rhythms will be entrained by an AM stimulus. However, open questions remain in how different rhythms echo after entrainment [32] and how this might impact the stimulus-evoked responses in their respective regions differently. Inhibition-timing views and communication through coherence views predict different outcomes in regard to which sources should show echoes in their oscillatory activity that more strongly parallel behavior, with the former necessitating relationships involving early auditory responses.

It may also be useful to explore the optimal frequency for entrainment effects on auditory working memory performance. It was chosen to use 8 Hz as the entrainment frequency here because of previous EEG work showing that prestimulus 8 Hz phase was predictive of performance in a pitch sequence identification task requiring tonal comparison [16] and a recent behavioral entrainment study showing optimal entrainment effects at 8 Hz [9]. However, a manipulation of a broader range of frequencies might help determine how well the optimal rate matches the eigenfrequencies of known brain rhythms that could be important for different aspects of the task, for example, lower alpha band oscillations for auditory “tau” rhythms [33,34,35] or frontal theta rhythms with correlates to working memory [36,37]. Further investigation into whether spontaneous prestimulus oscillations show relationships with auditory memory performance could also help answer the question as to whether a “rhythmic” processing mode introduced through entrainment is a prerequisite for the impacts of oscillations on auditory task performances [16,32].

As a final caveat, the design of experiments limited testing to only one potential cycle in an echo from entrainment. This allowed a sufficient number of trials to be collected within a single session. However, it also means that little can be said about how long entrainment effects last. A logical remedy to this would be to run a multisession experiment to collect enough trials at phase relationships with the entraining AM that span multiple cycles. Not only would this yield practical information regarding the use of entrainment for modulating human performance, but it would also be theoretically interesting to see whether or not behavioral memory effects of entrainment parallel neural entrainment effects (e.g., do they fade at the same pace?).

## 5. Conclusions

The current study shows that auditory AM-entraining sounds produce echoes in working memory for pitch. This shows up in cyclic modulations of performance in pitch matching, such that target sounds are more accurately remembered when they fall in phase with the entraining AM. The results open possibilities to extend the impacts of ongoing oscillatory brain states and entrainment further beyond its immediate perceptual consequences. They also lend support to proposals that ongoing oscillatory states modulate higher-order supramodal cognitive processes and point at a need to tease apart different functional roles.

## Data Availability

The raw data are available at https://osf.io/mkqgs/, accessed on 30 June 2024.
